# Exploring the phenotypic fingerprints of *ANXA11* variants in ALS: a population-based study in an European cohort

**DOI:** 10.1007/s00415-025-13276-w

**Published:** 2025-07-21

**Authors:** Francesca Palumbo, Barbara Iazzolino, Cristina Moglia, Umberto Manera, Enrico Matteoni, Sara Cabras, Maura Brunetti, Salvatore Gallone, Stefano Callegaro, Rosario Vasta, Gabriele Mora, Fabiola De Marchi, Lucia Corrado, Sandra D’Alfonso, Letizia Mazzini, Antonio Canosa, Maurizio Grassano, Andrea Calvo, Adriano Chiò

**Affiliations:** 1 ALS Center, ‘Rita Levi Montalcini’ Department of Neuroscience, University of Torino, Via Cherasco 15, 10126 Turin, Italy; 2 Neurology 1, Azienda Ospedaliero-Universitaria Città Della Salute E Della Scienza of Torino, Turin, Italy; 3 Institute of Cognitive Sciences and Technologies, National Research Council, Rome, Italy; 4 Department of Translational Medicine, University of Eastern Piedmont, Novara, Italy; 5 Division of Neurology, Azienda Ospedaliero-Universitaria Maggiore Della Carità, Novara, Italy; 6 Department of Health Sciences, University of Eastern Piedmont, Novara, Italy

**Keywords:** Amyotrophic lateral sclerosis, *ANXA11*, Phenotype, Cognition

## Abstract

**Background:**

*Annexin A11* (*ANXA11*) has emerged as a significant gene associated with amyotrophic lateral sclerosis (ALS) and cognitive impairments. This study aimed to evaluate the prevalence and clinical and cognitive features of pathogenic variants in *ANXA11* in an Italian ALS cohort.

**Methods:**

Data were collected from the Piemonte and Valle d’Aosta Register for ALS between 2009 and 2020. Only patients who underwent whole genome sequencing (WGS) were included. Clinical and cognitive assessments were compared among patients with *ANXA11*-ALS, wild-type ALS (WT-ALS), and *C9ORF72*-ALS.

**Results:**

Among 1,486 ALS patients, 18 (1.4%) were found to carry *ANXA11* variants, four of which were classified as benign or likely benign. Three patients (16.7%) also had co-occurring variants in *ERBB4* (*erb-b2 receptor tyrosine kinase 4*), *EPHA4* (*ephrin type-A receptor 4*), or *C9ORF72* (*chromosome 9 open reading frame 72*). Patients with ANXA11-ALS had significantly lower education levels (6.2 vs. 8.9 years), higher BMI at diagnosis (26.7 vs. 24.5), and a higher prevalence of cognitive impairment (100% vs. 47%) compared to WT-ALS. Cognitive testing revealed more severe deficits in executive function, attention, psychomotor speed, non-verbal intelligence, and cognitive flexibility, though no behavioral differences were observed. Compared to C9ORF72-ALS, ANXA11-ALS patients were older at diagnosis (66.6 vs. 60.3 years), had lower education levels (6.2 vs. 9.0 years), and higher rates of cognitive impairment (100% vs. 68.7%).

**Discussion:**

Pathogenic *ANXA11* variants are relatively common in ALS and are strongly associated with cognitive impairment. Including *ANXA11* in routine genetic screening may enhance diagnostic precision and therapeutic strategies for ALS patients.

**Supplementary Information:**

The online version contains supplementary material available at 10.1007/s00415-025-13276-w.

## Introduction

Amyotrophic lateral sclerosis (ALS) is a degenerative disorder of the central nervous system that affects upper and lower motor neurons and cortical neurons in the frontal and temporal lobes. It is characterized by progressive motor dysfunction and, in approximately 50% of cases, by cognitive and behavioral impairments, which can range from full-blown frontotemporal dementia (FTD) to milder cognitive alterations [1, 2]. Cognitive and behavioral dysfunctions are more commonly observed in patients carrying certain genetic mutations, particularly the GGGGCC repeat expansion in the first intron of the *C9ORF72* gene. Other genes that have been linked to an increased risk of dementia in ALS include *TARDBP, TBK1, VCP, MATR3, NEK1, C21ORF2,* and *TIA1* [3].

In 2017 genetic variants in the *vesicular trafficking protein annexin A11* (*ANXA11*) gene were reported to be associated with ALS, both with and without co-occurring FTD [4]. *ANXA11* is a 15 exon-gene located on chromosome 10q22.3 and encodes the 505 amino acid protein known as annexin A11 [5]. Pathogenic variants in *ANXA11* appear to be more frequent in Asia, accounting for 5 to 6% of familial ALS (FALS) cases and 2% of apparently sporadic ALS cases [6, 7]. In contrast, in Europe, *ANXA11* variants account for approximately 1% of ALS cases [8, 9]. Pathogenic *ANXA11* variants have also been reported in patients with FTD in the absence of motor neuron disease [10].

The clinical features of ALS patients carrying *ANXA11* variants remain poorly understood, and cognitive impairment in these patients has not yet been studied in detail.

The aim of this study is to characterize ALS patients with *ANXA11* variants (ANXA11-ALS) in an Italian population-based cohort, comparing them with ALS patients without pathogenetic variants of known ALS genes (WT-ALS) and those carrying the *C9ORF72* hexanucleotide expansion (C9ORF72-ALS).

## Methods

The study population consists of all ALS patients recorded in the prospective Piemonte and Valle d’Aosta Register for ALS (PARALS) between 2009 and 2020. PARALS is a prospective, population-based register that has been active in two regions of Northern Italy since 1995. The detailed characteristics of the register have been described elsewhere [11]. For this study, only patients who underwent whole-genome sequencing (WGS) were included. ALS diagnoses were established according to the Gold Coast criteria [12].

At time of diagnosis, we collected demographic and clinical data of patients, including the ALS Functional Rating Scale—Revised (ALSFRS-R). ALSFRS-R mean monthly decline (∆ALSFRS-R) was calculated using the following formula: (48—*ALSFRS-R score at diagnosis*)/(*months from onset to diagnosis*). Similarly, weight mean monthly decline (∆Weight) was calculated as (*Weight at diagnosis*—*healthy body weight*)/*months from onset to diagnosis*.

Patients with a history of disorders that may potentially affect cognition (i.e. mental retardation, major stroke, severe head injuries), alcohol or drug dependence, severe mental illness, or use of high-dose psychoactive medications were not included in data analysis. Patients who were not of native Italian language were assessed only through an unstructured interview and were excluded from the analysis. A total of 134 age- and sex-matched controls were also tested with the same battery. Control subjects were recruited among residents in retirement homes or non-consanguineous relatives of cases.

### Neuropsychological assessment and domain classification of tests 

Patients and controls underwent a battery of neuropsychological tests encompassing executive function, verbal and visual memory, attention and working memory, visuospatial function, language, social cognition, and behavior. The battery of tests was administered within 2 months from diagnosis. The tests were selected according to the Diagnostic Criteria for the Behavioural variant of Frontotemporal Dementia [13], and ALS-FTD Consensus Criteria (ALSFTD-CC) [14]. The list of tests and their classification according to the main neuropsychological domain is reported in the Supplementary Methods and the Supplementary Tables 1 and 2 [15–17].

According to the ALSFTD-CC [14] patients were classified into five categories: (1) patients with normal cognition (ALS-CN); (2) patients with isolated cognitive impairment (ALSci), i.e., patients with evidence of executive and/or language dysfunction. Executive impairment was defined as impaired verbal fluency (letter) and/or impairment on two other non-overlapping measures of executive functions; (3) patients with isolated behavioral impairment (ALSbi), characterized by apathy with or without other behavioral changes; (4) patients with both cognitive and behavioral impairment (ALScbi), meeting the criteria for both ALSci and ALSbi; and (5) patients with frontotemporal dementia (ALS-FTD).

### Genetic screening 

A total of 1486 patients underwent whole genome sequencing (WGS). WGS methodology and quality control filters are reported in detail elsewhere [18] and are summarized in the Supplementary methods. Pathogenic variants of 46 ALS-related genes were extracted [18]. Variants classification was based on the 2015 ACMG-AMP (American College of Medical Genetics and Genomics—Association for Molecular Pathology) guidelines [19].

Loss-of-function and previously reported ALS variants were considered pathogenic unless present in the control cohort. The remaining rare variants (defined as minor allele frequency less than 0.0001 in the non-Finnish European population) were then classified based on computational prediction and expert review as reported in Supplementary Methods and included if deemed to be potentially pathogenic (Supplementary Fig. 1).

All patients were screened by a repeat-primed PCR assay for the presence of the GGGGCC hexanucleotide expansion in the first intron of *C9ORF72* [20]. Repeat lengths of ≥ 30 units with the characteristic sawtooth pattern were considered to be pathogenic [20].

### Statistical methods 

Comparisons between tests were conducted on age-, sex-, and education-corrected scores. Due to non-normal distribution in most cognitive test scores, the Mann–Whitney U test was used for comparisons. Considering the difference of age, sex and site of onset between ANXA11-ALS, patients without pathogenetic variants of 46 ALS-related genes (WT-ALS), patients with *C9ORF72* GGGGCC repeat expansion (C9ORF72-ALS) and controls, we compared ANXA11-ALS with the other groups balancing for these predictor variables using propensity score matching as implemented in the R "Matchit" package (version 4.1.0). We performed a 3:1 (with WT-ALS), 2:1 (with controls) and 2:1 (with C9ORF72-ALS) nearest neighbor matching without replacement using logistic regression to calculate propensity scores. The resulting groups were balanced for all variables. Cohen’s effect sizes were also reported. All analyses were carried out with SPSS 29.0 statistical package (SPSS, Chicago, IL, USA).

### Standard protocol approvals, registrations, and patient consents 

The study was approved by the Ethics Committee of the ALS Expert Center of Torino (Comitato Etico Azienda Ospedaliero-Universitaria Città della Salute e della Scienza, Torino, #0036344, #0038876 and #0064510). Patients and controls provided written informed consent before enrollment. The database was anonymized according to Italian law for the protection of privacy.

## Results

**Fig. 1:**
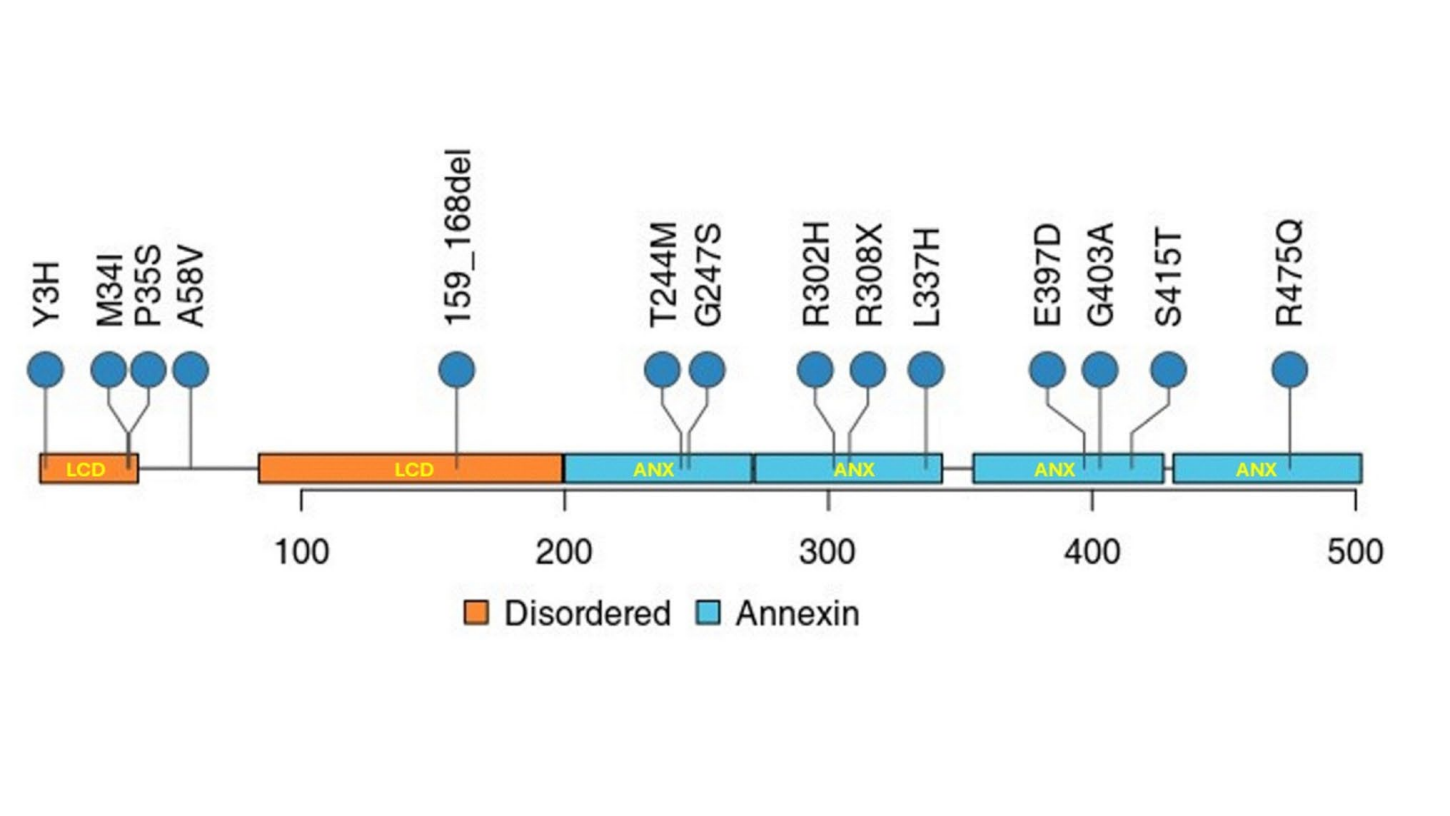
Schematics of the protein domain structure of *ANXA11* and identified variants. LCD, low-complexity domain; ANX, ANX domain

The flow-chart of the study is reported in Supplementary Fig. 1. A total of 1,758 incident ALS patients were included in the PARALS registry between 2009 and 2020, of whom 1,486 (84.5%) underwent whole-genome sequencing (WGS). Three hundred sixty-five patients carried pathogenic variants in genes other than *ANXA11* or *C9ORF72* and were excluded from this study. Eighteen patients (1.2%) carried pathogenic or putative deleterious variants in the *ANXA11* gene (Fig. 1). The list of *ANXA11* pathogenic variants is provided in Supplementary Table 3. We found a total of 13 novel variants (transcript NM_00157): p.Y3H (c.7A > G), p.M34I (c.102G > A) in two apparently unrelated cases, p.P35S (c.103C > T), p.A58V (c.173C > T), p.T244M (c.731C > T), p.G247S (c.739G > A), p.R302H (c.905G > A), p.R308X (c.823G > A), p.L337H (c.1010A > T), p.E397D (c.1191G > C), p.G403A (c.1208G > C), p.S415T (c.1243 T > A) and p.R475Q (c.1424G > A). Of these, p.Y3H (c.7A > G), p.T244M (c.731C > T), p.R302H (c.905G > A), and p.L337H (c.1010A > T) were classified as Benign or Likely Benign by VarSome and were excluded from the subsequent analyses. In addition, we found a 10 amino acids deletion p.159_168del (c.477_503del) in one patient. Gene predictions of novel *ANXA11* variants are reported in Supplementary Table 4. Notably, three patients had a second genetic variant besides *ANXA11*: one patient carried both an *ANXA11* variant (p.R308X) and an *ERBB4 (erb-b2 receptor tyrosine kinase 4*) gene variant (p.E581G), one patient carried both an *ANXA11* variant (p.L337H) and an *EPHA4* (*ephrin type-A receptor 4*) gene variant (p.S687C), and one patients carried both an *ANXA11* pathogenic variant (p.S415T) and a *C9ORF72* GGGGCC repeat expansion. Due to the strong phenotypic imprint of *C9ORF72* gene, the latter patient was excluded from further analyses.

### Clinical characteristics of ANXA11 patients 

The clinical and demographic characteristics of the 14 ANXA11-ALS cases, compared to 1010 WT-ALS and 93 C9ORF72-ALS, are reported in Table 1.

**Table 1:** Clinical and demographic characteristics of ANXA11-ALS compared to WT-ALS and C9ORF72-ALS

	ANXA11-ALS N = 14	WT-ALS N = 1010	C9ORF72-ALS N = 93	p-value (ANXA11-ALS vs WT-ALS)	p-value (ANXA11-ALS vs C9ORF72-ALS)
Sex (male, %)	10 (71.4%)	563 (55.7%)	46 (49.5%)	0.37	0.21
Mean age at onset (years, SD)	66.6 (10.0)	66.9 (11.0)	60.3 (8.9)	0.79	**0.0001**
Mean onset-diagnosis time (months, SD)	12.4 (8.1)	12.5 (11.9)	9.6 (6.1)	0.97	0.109
Mean education years (SD)	6.2 (2.4)	8.9 (4.1)	9.0 (3.6)	**0.006**	**0.002**
Site of onset (Bulbar, %)	4 (28.6%)	314 (31.1%)	39 (41.9%)	0.84	0.51
Mean ALSFRSr at diagnosis (SD)	38.5 (6.4)	39.3 (7.1)	40.6 (6.6)	0.67	0.24
Mean ALSFRSr decline (points/month)	1.15 (1.31)	1.21 (1.74)	1.11 (1.52)	0.89	0.90
King’s Staging (1/2/3/4)	4/4/6/0	361/337/277/36	39/30/22/2	0.86	0.69
King’s decline (points/months)	0.30 (0.36)	0.29 (0.31)	0.30 (0.33)	0.90	0.96
MiToS Staging (0/1/2/3/4)	7/7/0/0/0	646/318/36/9/2	66/25/1/1	0.88	0.64
Cognitively impaired at diagnosis (N, %) °*	12 (100%)	348 (47.0%)	57 (68.7%)	**0.0008**	**0.01**
FALS	4 (28.5%)	57 (5.6%)	59 (63.4%)	**0.0001**	**0.03**
Mean BMI (premorbid, SD)	27.8 (4.2)	26.0 (4.1)	25.5 (5.1)	0.073	0.087
Mean BMI (at diagnosis, SD)	26.7 (4.3)	24.5 (4.2)	24.2 (4.9)	**0.032**	**0.046**
Mean weight decline (Kg/month) +	0.61 (0.89)	0.66 (1.08)	0.47 (1.00)	0.86	0.61
Mean FVC% at diagnosis, SD) #	81.3 (26.6)	86.9 (27.3)	82.6 (29.3)	0.41	0.87
Median survival (years, SD)	4.3 (2.2–7.6)	2.7 (1.7–5.2)	2.5 (1.7–3.4)	0.46	**0.035**

° ALS-CN vs ALSci + ALSbi + ALScbi + ALS-FTD

^*^Cognition available in: ANXA11-ALS, 12; WT-ALS, 740; C9ORF72-ALS, 83

^#^ FVC available in: ANXA11-ALS, 14; WT-ALS, 903; C9ORF72-ALS, 87

+ Weight available in: ANXA11, 14; WT-ALS, 987; C9ORF72-ALS, 92

Compared to WT-ALS, ANXA11-ALS cases had a lower mean education year (6.2 [SD 2.4] vs 8.9 [SD 4.1), p = 0.006) and a higher mean BMI at diagnosis (26.7 [SD 4.3] vs 24.5 [SD 4.2], p = 0.032). ANXA11 patients were more frequently cognitively impaired (100% vs 47%, p = 0.0008) and had more frequently a family history for ALS/FTD (28.5% vs 5.6%, p = 0.0001). In addition, ANXA11-ALS cases had a longer median survival (4.3 years [IQR 2.2–7.6] vs. 2.7 years [IQR 1.7–5.2]), though this difference was not statistically significant (p = 0.46).

Compared to C9ORF72-ALS, ANXA11-ALS had a higher mean age at onset (66.6 [SD 10.0] year vs 60.3 [8.9] years, p = 0.0001), lower mean education years (6.2 [SD 2.4] vs 9.0 [SD 3.6), p = 0.002) and a higher mean BMI at diagnosis (26.7 [SD 4.3] vs 24.2 [SD 4.9], p = 0.046). ANXA11-ALS patients were more cognitively impaired (100% vs 68.7%, p = 0.01) and had less frequently a family history for ALS/FTD (28.5% vs 63.4%, p = 0.03). They had also a longer median survival (4.3 years [IQR 2.2–7.6] vs. 2.5 years [IQR 1.7–3.4], p = 0.035).

### Detailed analysis of cognitive performance of ANXA11-ALS 

A comprehensive cognitive analysis was available for 12 ANXA11-ALS, 74 C9ORF72-ALS, and 730 WT-ALS. All ANXA11-ALS had cognitive impairment compared to 49% of WT-ALS and 60.9% of C9ORF72-ALS (Table 2).

**Table 2:** Cognitive classification of ANXA11-ALS, C9ORF72-ALS (p = 0.011), and WT-ALS (p = 0.0001)

	#	ALS-CN	ALSci	ALSbi	ALScbi	ALS-FTD
*ANXA11-ALS*	12	0	4 (33.3%)	2 (16.7%)	1 (8.4%)	5 (41.6%)
*C9ORF72-ALS*	74	29 (39.2%)	16 (21.6%)	6 (8.1%)	6 (8.1%)	17 (23.0%)
*WT-ALS*	730	378 (51%)	145 (19.9%)	83 (11.4%)	64 (8.8%)	60 (8.2%)

To identify the specific cognitive tests and domains impaired in ANXA11-ALS, we initially compared ANXA11-ALS patients with healthy controls (HC) in a 1:2 ratio, balanced using propensity scores as described in the Methods section. ANXA11-ALS patients performed worse on nearly all tests, except for Trail Making Test A (TMT A), Rey Auditory Verbal Learning Test—Delayed Recall (RAVL-DR), Babcock Story Recall Test, Immediate Recall (BSRT-IR) and delayed recall (BSRT-DR). No significant differences were observed for Hospital Anxiety and Depression Scale (HADS-A or HADS-D) (Table 3).

**Table 3:** Comparison of cognitive tests in ANXA-ALS vs HC

	ANXA11 n = 12	HC n = 24	p-value
MMSE	26.7 (24.0–29.9) N = 12	30 (28.0–30.0) N = 24	**0.011**
FAS	21.4 (14.6–26.6) N = 12	32.7 (27.4–39.5) N = 24	**0.0001**
CAT	15.4 (12.5–19.1) N = 12	19.5 (17.3–22.6) N = 24	**0.015**
FAB	13.5 (11.6–15.5) N = 11	18.0 (16.5–18.0) N = 24	**0.0001**
Digit Span FW	4.4 (4.1–4.9) N = 10	6.5 (6.0–7.2) N = 24	**0.0001**
Digit Span BW	3.7 (3.0–4.1) N = 10	4.9 (4.1–5.9) N = 24	**0.003**
TMT A	44 (28–110) N = 10	38.0 (25.2–49.8) N = 24	0.152
TMT B	102 (94–300) N = 10	63 (35–86) N = 24	**0.003**
TMT B-A	76 (39–204) N = 10	17.5 (5–53) N = 24	**0.001**
RAVL-IR	33.0 (27.5–43) N = 8	46.9 (39.4–51.4) N = 24	**0.042**
RAVL-DR	4.4 (2.1–10.4) N = 8	8.7 (8.0–11.8) N = 24	0.112
BSRT-IR	4.5 (3.4–6.2) N = 8	6.0 (5.4–7.0) N = 24	0.106
BSRT-DR	5.3 (2.9–7.3) N = 8	6.4 (6.0–6.9) N = 24	0.494
ROCF-IR	28.5 (17.8–31.6) N = 8	35.5 (31.56–36.0) N = 24	**0.022**
ROCF-DR	7.3 (6.3–10.0) N = 8	18.0 (14.0–21.0) N = 24	**0.001**
Clock	3 (2.5–4) N = 12	5 (4–5) N = 24	**0.0001**
CPM47	25.0 (23.5–29.0) N = 11	32.1 (30.0–35.4) N = 24	**0.0001**
HADS-A	5.0 (4.5–14.0) N = 12	6.0 (4.3–7.0) N = 24	0.809
HADS-D	5.5 (1.0–9.5) N = 12	2 (1.2–6.0) N = 24	0.223

The comparison of ANXA11-ALS with age-, education- and sex-matched WT-ALS (with a proportion of 1:3) showed that ANXA11-ALS had worse performances in Letter Fluency test (FAS), Category Fluency Test (CAT), Frontal Assessment Battery (FAB), Digit Span Forward (FW), TMT A, TMT B, TMT B-A, Raven’s Colored Progressive Matrices (CMP47), and Clock test (Table 4).

**Table 4:** Comparison of cognitive tests between ANXA-ALS and WT-ALS

	ANXA11-ALS n = 12	WT-ALS n = 36	p-value
MMSE	26.7 (24.0–29.9) N = 12	27.8 (25.8–30.0) N = 36	0.400
FAS	21.4 (14.6–26.6) N = 12	28.6 (22.4–39.4) N = 36	**0.009**
CAT	15.4 (12.5–19.1) N = 12	19.8 (17.7–22.0) N = 36	**0.025**
FAB	13.5 (11.6–15.5) N = 11	16.1 (14.3–18.0) N = 34	**0.004**
Digit Span FW	4.4 (4.1–4.9) N = 10	5.7 (5.2–6.2) N = 32	**0.0001**
Digit Span BW	3.7 (3.0–4.1) N = 10	4.1 (3.6–4.7) N = 32	0.107
TMT A	44 (28–110) N = 10	33.0 (19.0–50.0) N = 32	**0.037**
TMT B	102 (94–300) N = 10	44 (22–127) N = 32	**0.017**
TMT B-A	76 (39–204) N = 10	25 (3–82) N = 32	**0.045**
RAVL-IR	33.0 (27.5–43) N = 8	38.2 (33.7–45.5) N = 26	0.193
RAVL-DR	4.4 (2.1–10.4) N = 8	7.2 (5.1–8.7) N = 26	0.318
BSRT-IR	4.5 (3.4–6.2) N = 8	4.8 (3.5–7.2) N = 26	0.651
BSRT-DR	5.3 (2.9–7.3) N = 8	6.3 (4.5–7.2) N = 26	0.475
ROCF-IR	28.5 (17.8–31.6) N = 8	31.5 (28.6–33.5) N = 26	0.207
ROCF-DR	7.3 (6.3–10.0) N = 8	10.3 (7.5–14.5) N = 26	0.207
Clock	3 (2.5–4) N = 12	5 (3–5) N = 36	**0.027**
CPM47	25.0 (23.5–29.0) N = 11	29.3 (24.9–32.6) N = 36	**0.045**
HADS-A	5.0 (4.5–14.0) N = 12	8.0 (5.0–12.0) N = 35	0.687
HADS-D	5.5 (1.0–9.5) N = 12	4.0 (2.0–8.0) N = 35	0.870

In the comparison of ANXA11-ALS with age-, education- and sex-matched C9ORF72-ALS (with a proportion of 1:2), the only test that was significantly more impaired in ANXA11-ALS was Digit Span Forward (FW), while no test was more impaired in C9ORF72-ALS (Table 5).

**Table 5:** Comparison of cognitive tests between ANXA-ALS and C9ORF72-ALS

	ANXA11-ALS n = 12	C9ORF72-ALS n = 24	p-value
MMSE	26.7 (24.0–29.9) N = 12	27.5 (26.5–29.5) N = 24	0.548
FAS	21.4 (14.6–26.6) N = 12	23.4 (16.4–32.3) N = 24	0.310
CAT	15.4 (12.5–19.1) N = 12	17.3 (12.43–20.6) N = 24	0.664
FAB	13.5 (11.6–15.5) N = 11	14.7 (12.6–17.0) N = 24	0.727
Digit Span FW	4.4 (4.1–4.9) N = 10	5.1 (4.5–5.9) N = 24	**0.011**
Digit Span BW	3.7 (3.0–4.1) N = 10	3.9 (3.3–4.1) N = 24	0.696
TMT A	44 (28–110) N = 10	37.0 (25.3–56.0) N = 24	0.169
TMT B	102 (94–300) N = 10	80.5 (33.5–242.5) N = 24	0.197
TMT B-A	76 (39–204) N = 10	35.0 (8.0–154.5) N = 24	0.212
RAVL-IR	33.0 (27.5–43) N = 8	29.3 (31.0–39.5) N = 16	0.441
RAVL-DR	4.4 (2.1–10.4) N = 8	6.4 (4.9–7.5) N = 16	0.594
BSRT-IR	4.5 (3.4–6.2) N = 8	6.1 (3.4–6.5) N = 16	0.601
BSRT-DR	5.3 (2.9–7.3) N = 8	6.0 (2.5–7.0) N = 16	1.000
ROCF-IR	28.5 (17.8–31.6) N = 8	31.1 (27.0–34.1) N = 22	0.333
ROCF-DR	7.3 (6.3–10.0) N = 8	11.5 (7.2–15.3) N = 22	0.423
Clock	3 (2.5–4) N = 12	4 (3–5) N = 21	0.305
CPM47	25.0 (23.5–29.0) N = 11	31.4 (18.7–32.7) N = 26	0.336
HADS-A	5.0 (4.5–14.0) N = 12	5.0 (3.8–9.2) N = 24	0.408
HADS-D	5.5 (1.0–9.5) N = 12	4.5 (1.0–7.3) N = 24	0.588

### Correlation between the localization of the pathogenic variants and the clinical characteristics of patients 

Eight patients had variants in the N-terminal Low Complexity Domain (LCD) region of the protein, while six had variants in the C-terminal (non-LCD) region (Table 6). The two groups did not differ significantly across any of the variables analyzed. Specifically, the age at onset was 66.6 years (SD 7.8) in the LCD cases and 66.5 (SD 13.3) in non-LCD cases (p = 0.76) and the median survival time was 4.3 (IQR 2.3–7.6) in the LCD cases and 2.9 (2.1–6.1) in the non-LCD cases (p = 0.49). Three patients out of the 8 tested in the LCD group had FTD (37.5%) compared to 2 out of the 6 tested in the non-LCD group (33.3%) (p = 0.29).

**Table 6:** Characteristics of patients carrying ANXA11 pathogenic variants according to the genetic location of the variants

	LCD (n = 8)	Non-LCD (n = 6)	p-value
Sex (female, %)	2 (25.0%)	1 (16.7%)	0.14
Mean age at onset (mean, SD)	66.6 (7.8)	66.5 (13.3)	0.76
Site of onset (bulbar, %)	1 (12.5%)	3 (50%)	0.19
Diagnostic delay (months, SD)	12.6 (7.6)	12.1 (9.2)	0.92
FTD	3 (37.5%)	2 (33.3%)	0.29
fALS (%)	1 (12.5%)	2 (33.3%)	0.45
Mean ALSFSR-R score (SD)	39.0 (6.7)	38 (6.5)	0.76
∆ALSFRS-R (points/month)	0.82 (0.52)	1.54 (182)	0.27
∆Weight (kg/month)	0.50 (0.69)	0.73 (1.10)	0.61
Median Survival (years)	4.3 (2.3–7.6)	2.9 (2.1–6.1)	0.49

*LCD* low complexity domain, Non-LCD, other domains

## Discussion

We have described a series of ALS patients carrying pathogenic variants in the *ANXA11* gene. In our population-based epidemiological cohort, ANXA11-ALS accounted for 1.0% of cases, making *ANXA11* one of the most commonly identified ALS-associated genes [21]. Notably 9 pathogenic variants were previously not reported. ANXA11-ALS presents a distinctive phenotype, characterized by a high co-morbidity with FTD, later age at onset, higher BMI at diagnosis, and lower educational level compared to WT-ALS and C9ORF72-ALS patients. Although ANXA11-ALS is associated with several negative prognostic factors, it shows better survival outcomes than both WT-ALS and C9ORF72-ALS.

The most notable finding in our series of ANXA11-ALS patients is that all individuals who underwent formal cognitive assessment at diagnosis exhibited some degree of cognitive impairment. This aligns with previous studies reporting a strong association between *ANXA11* and co-morbid FTD in ALS [8, 22, 23], although not all studies have confirmed this link [24]. The consistent cognitive impairment observed in ANXA11-ALS patients indicates that genetic screening for ANXA11 variants should be prioritized in ALS patients with cognitive symptoms, while comprehensive neuropsychological evaluation must be performed in all identified ANXA11 carriers. This clear genotype–phenotype correlation also directly facilitate variant interpretation and enables more precise genetic counseling for patients and their families.

In our study, we conducted a more detailed analysis of cognitive impairment in ANXA11-ALS patients, revealing that the deficits are more severe than those observed in WT-ALS, particularly in tests assessing executive function (FAS, CAT, FAB, TMT B-A, Clock Drawing), as well as attention/working memory (Digit Span Forward), psychomotor speed (TMT-A), non-verbal intelligence (CPM-47), and cognitive flexibility (TMT-B). No significant differences were found in the behavioral domain, as apathy emerged as the most common symptom in both ANXA11-ALS and WT-ALS patients.

To the best of our knowledge, no studies have directly compared cognitive performance between C9ORF72-ALS and ANXA11-ALS. In our cohort, ANXA11-ALS patients exhibited a cognitive and behavioral profile broadly similar to that of C9ORF72-ALS. The only statistically significant difference was poorer performance on the Digit Span Forward test, which primarily assesses attention and working memory. However, when considering the ALS-FTD-CC cognitive/behavioral classification at the time of diagnosis, ANXA11-ALS patients appeared to be more cognitively impaired than those with C9ORF72-ALS. Specifically, 39.2% of C9ORF72-ALS patients were cognitively normal, whereas none of the ANXA11-ALS patients met criteria for cognitive normality. Moreover, 41.6% of ANXA11-ALS cases were classified as ALS-FTD, compared to 23% of C9ORF72-ALS cases. Although these findings require replication in independent cohorts, they suggest that ANXA11-ALS may be associated with a greater propensity for early cognitive and behavioral impairment.

The observation that ANXA11-ALS patients in our series had a significantly lower level of education compared to both WT-ALS and C9ORF72-ALS is noteworthy. Education level is considered a proxy for cognitive reserve, a key factor mediating cognitive impairment in both Alzheimer’s disease [25, 26], and FTD, with or without ALS [27–29]. Unfortunately, education level has been largely overlooked in studies on *ANXA11* [23, 30, 31]. One exception is a report showing that patients with pathogenic variants of *ANXA11* had a significantly lower education level (5.3 years) than those with non-pathogenic variants (9.4 years) [32].

Our propensity score matching approach accounted for education level as a potential confounding variable, thereby strengthening the evidence for a direct association between the ANXA11 genotype and cognitive phenotype. However, we cannot exclude the possibility that the lower education levels observed in ANXA11 carriers may themselves reflect the early cognitive effects of these genetic variants.

These findings should be interpreted within the broader framework of the bidirectional relationship between education and cognitive function. On one hand, lower cognitive abilities in early life—whether driven by genetic, developmental, or environmental factors—may hinder academic achievement and limit access to higher levels of education. Subtle cognitive impairments during childhood or adolescence can negatively impact school performance, ultimately resulting in reduced educational attainment. On the other hand, educational attainment is a well-established determinant of long-term cognitive health. It is a major contributor to cognitive reserve, the brain’s capacity to compensate for neurodegenerative pathology. Higher levels of education are associated with greater resilience to cognitive decline, whereas lower educational attainment may reduce this reserve and increase vulnerability to early clinical manifestations of neurodegenerative diseases. Thus, when evaluating cognitive performance in patients with neurodegenerative disorders, it is crucial to consider education not only as a potential confounder but also as a meaningful modifying factor that may shape the clinical expression of disease.

*ANXA11* has also been implicated in pure FTD cases. In a Chinese cohort of 261 FTD patients, *ANXA11* variants were identified in 4 individuals (1.5%), making it the fifth most common gene associated with FTD, following *MAPT, TBK1, OPTN,* and *GRN* [10]. Additionally, a case of FTD with an *ANXA11* variant (p.D40G) was reported in a study of 29 sporadic FTLD patients [33]. Another eight patients with various *ANXA11* variants were identified by Jang et al. [32].

Our findings, together with previous reports, firmly establish ANXA11 as a significant contributor to the continuum of motor neuron and frontotemporal syndromes.

In our study, we could not confirm a previous observation that *ANXA11* variants in the N-terminal LCD region are associated with later age at onset, higher ΔALSFRS-R, and a higher frequency of FTD compared to variants in the non-LCD region [30]. The differing ethnic backgrounds between our cohort and that study may explain these discrepancies. However, the small sample sizes in both studies warrant cautious interpretation of these divergent findings.

Interestingly, in three patients from our series (16.7%), including one carrying the likely benign variant p.L337H, we identified the co-occurrence of an *ANXA11* variant along with variants in the *ERBB4* and *EPHA4* genes, as well as a *C9ORF72* GGGGCC repeat expansion. Similar findings have been reported in other studies, which identified patients with *ANXA11* variants co-occurring with ANK-binding kinase 1 *(TBK1)* (p.R143C), *GLE1 RNA Export Mediator (GLE1)* (p.T620A) [8], and TAR DNA-binding protein 43 *(TARDBP)* (p.N352S, in two patients) [24].

*ANXA11* appears to be a pleiotropic gene, as pathogenic variants have been associated with a range of diverse clinical presentations, including corticobasal syndrome [34], childhood-onset oculopharyngeal muscular dystrophy [35], inclusion body myopathy with predominant limb-girdle syndrome [36], semantic variant primary progressive aphasia [37], and ALS with progressive supranuclear features [38]. The pleiotropic behavior of *ANXA11* is comparable to that observed in other ALS-related genes, such as *Valosin Containing Protein* (*VCP)* and Matrin 3 (*MATR3)* [39].

This study is not without limitations. First, not all cases underwent extensive cognitive testing; however, the non-tested cases did not differ clinically from those that were tested, which reduces the risk of selection bias. Second, while the cohort of ANXA11-ALS patients is relatively small, it represents a complete set of individuals identified through a prospective epidemiological register, providing reliable estimates of the frequency of ANXA11 pathogenic variants in Italy. Third, the number of age- and sex-matched controls is relatively small. However, in the analyses, controls were matched to ANXA11-ALS cases at a 2:1 ratio using propensity score matching.

In conclusion, this population-based study from an Italian epidemiological register demonstrates that ANXA11 pathogenic variants account for approximately 1.0% of ALS cases, with all tested individuals exhibiting some degree of cognitive and behavioral impairment. Our findings have implications for clinical practice, highlighting that *ANXA11* is not uncommon in the ALS population and represents one of the most frequently implicated genes in cognitive co-morbidity.

## Supplementary Information

Below is the link to the electronic supplementary material.

Supplementary file1 (DOCX 124 KB)

## Data Availability

Anonymised data relating to this article will be made available by request from any qualified investigator, subject to approval from the Comitato Etico Azienda Ospedaliero-Universitaria Città della Salute e della Scienza.
