# Supplementary material for: Exploring the phenotypic fingerprints of ANXA11 variants in ALS: a population-based study in an European cohort
Source: J Neurol. 2025 Jul 21;272(8):524. doi: 10.1007/s00415-025-13276-w (PMC12279584; doi:10.1007/s00415-025-13276-w)
Supplement: Supplementary file 1 — Supplementary file1 (DOCX 124 KB) [file 415_2025_13276_MOESM1_ESM.docx]

**Supplementary methods**

***List of performed tests***. Executive function was tested with Letter Fluency test (FAS), Category Fluency Test (CAT), Trail Making Test B-A (TMT B-A), Frontal Assessment Battery (FAB), ECAS Executive Function score, and ECAS Verbal Fluency score; Language was assessed with ECAS Language subscore and Boston Naming Test; Verbal Memory with Rey Auditory Verbal Learning Test, Immediate Recall (RAVL-IR), Rey Auditory Verbal Learning Test, delayed Recall (RAVL-DR), Babcock Story Recall Test, Immediate Recall (BSRT-IR), Babcock Story Recall Test, Delayed Recall (BSRT-DR), and ECAS Memory score; Visual Memory with Rey-Osterrieth Complex Figure Test, differed recall (ROCF-DR); Visuoconstructive abilities with Rey-Osterrieth Complex Figure Test, Immediate Recall (ROCF-IR), Clock Drawing Test (Clock), and ECAS Visuospatial Abilities score; Attention/Working memory with Digit Span Forward (FW) and Digit Span Backward (BW); Psychomotor speed with Trail Making Test A (TMT A); Non-Verbal Intelligence with Raven’s Colored Progressive Matrices (CPM47); Cognitive flexibility with Trail Making Test B (TMT B); Theory of Mind (social cognition) with Story-based Empathy Task (SET); Behavior with Frontal Systems Behavior Scale (FrSBe), ECAS, and Frontal Behavioural Inventory (FBI). Neurobehavioral dysfunction was determined with the Frontal Systems Behaviour Scale (FrSBe), using the Family-form evaluated by a close relative/caregiver (scores: normal ≤59, borderline 60-64; pathological ≥65). For the purpose of this study, we considered the change in points for each of the 3 domains of FrSBe (apathy, disinhibition, executive) from the before disease to the disease scores. If a subject had scores reflecting a frontal systems abnormality both in the premorbid and in the post-illness forms, he/she was considered pathological only if there was an increase of ≥10 points at the T-score between the two forms (Montuschi et al, 2015).

For ECAS we used the validated Italian version (Poletti et al 2018), and for the behavioral component of ECAS the cut-off based on number of symptoms (Poletti et al 2023).

**Whole-genome sequencing**

Whole-genome sequencing was performed at The American Genome Center at the Uniformed Services University on the Walter Reed National Military Medical Center campus in Bethesda, MD, USA. Briefly, libraries were prepared using TruSeq DNA PCR-Free High Throughput Library Prep Kit (Illumina Inc.) as per the manufacturer's instructions. Sequencing was performed on an Illumina HiSeq X10 sequencer using pairedend 150 base pair reads, and the data were processed according to Genome Analysis Toolkit's (GATK) best practices (https://software.broadinstitute.org/gatk/best-practices/). Variant quality control was performed using the GATK variant quality score method with default filters using Genome Reference Consortium Human Build 38 as the reference

**ALS-related genes**

We extracted variant information from the data for the following genes: *ALS2, ANXA11, ATXN2, C21orf2, C9orf72, CCNF, CHCHD10, CHMP2B, DAO, DCTN1, DNAJC7, ERBB4, EWSR1, FIG4, FUS, GLE1, GRN, HRNPA1, HRNPA2B1, HNRNPD, KIF5A, MAPT, MATR3, NEFH, NEK1, OPTN, PFN1, PRNP, PRPH, SETX, SIGMAR1, SOD1, SPG11, SPTLC1, SQSTM1, SS18L1, TAF15, TARDBP, TBK1, TUBA4A, UBQLN2, VAPB,* and *VCP*.

**Variant annotation**

Annotation was then performed using ANNOVAR v2020-06-07 (https://annovar.openbioinformatics.org) and KGGseq v1.0 (http://pmglab.top/kggseq/) using the gnomAD database (version 2.1.1) to determine minor allele frequency (MAF) in the European-derived population. The current study was focused on coding variants due to the limitations in interpreting non-coding variants.

**Expansion screening**

The samples were screened for *C9orf72* expansions using repeat-primed PCR methodology as previously described (Renton et al, 2011). A cut-off of 30 repeated expansion and the characteristic sawtooth pattern was considered pathogenic for *C9orf72*2. ExpansionHunter - Targeted software (version 0.3) was used to estimate repeat lengths of known, disease-causing expansions in samples undergoing whole-genome sequencing. This algorithm has been validated using experimentally confirmed samples carrying expansions (Dolzhenko et al, 2017). The performance of whole-genome sequencing in terms of sensitivity for repeated expansion has been validated in the same ALS cohort (Grassano et al, 2020; Grassano et al, 2022).

**Variant interpretation**

We set a conservative MAF frequency threshold of <0.01% based on the epidemiology of ALS. We defined Loss of Function (LoF) variants when the sequence changes were predicted to be a premature stop codon, a frameshift causing insertion/deletion (indel), or a splice-site disrupting variant located in the canonical splice sites (+1 and +2, -1 and -2) that cause the premature termination codon falling < 50-55 nucleotides upstream of the 3’ most exon–exon junctions. Loss of function variants was considered deleterious unless they exceeded the 0.01% MAF threshold. Variants previously reported in ALS or classified as Pathogenic or Likely Pathogenic according to the American College of Medical Genetics (ACMG) Guidelines for variant interpretation and classification were deemed as pathogenic. Benign or Likely Benign variants were excluded from the analysis. For all other type (Variants of Uncertain Significance, VUS) of variants, the combination of different sets of algorithms was considered using recommended threshold [MutationTaster, Combined Annotation Dependent Depletion (CADD), Sorting Intolerant From Tolerant (SIFT), Polymorphism Phenotyping (PolyPhen),Protein Variation Effect Analyzer (PROVEAN), Functional Analysis through Hidden Markov Models (Fathmm-MKL)] (Anderson & Lassmann, 2018). Those variants underwent an independent review by clinical and genetic ALS experts and were confirmed to be clinically reportable if agreed by consensus. When available, gene-specific information data was used in the classification

**Data availability statement**

The individual-level sequence data are available on dbGaP (accession number: phs001963.v1.p1) upon motivated request by interested researchers.

**Supplementary references**

Anderson D, Lassmann T. A phenotype centric benchmark of variant prioritisation tools. npj Genom Med. 2018;3(1):5. doi:10.1038/s41525-018-0044-9

Dolzhenko E, van Vugt JJFA, Shaw RJ, Bekritsky MA, van Blitterswijk M, Narzisi G, Ajay SS, Rajan V, Lajoie BR, Johnson NH, Kingsbury Z, Humphray SJ, Schellevis RD, Brands WJ, Baker M, Rademakers R, Kooyman M, Tazelaar GHP, van Es MA, McLaughlin R, Sproviero W, Shatunov A, Jones A, Al Khleifat A, Pittman A, Morgan S, Hardiman O, Al-Chalabi A, Shaw C, Smith B, Neo EJ, Morrison K, Shaw PJ, Reeves C, Winterkorn L, Wexler NS; US–Venezuela Collaborative Research Group; Housman DE, Ng CW, Li AL, Taft RJ, van den Berg LH, Bentley DR, Veldink JH, Eberle MA. Detection of long repeat expansions from PCR-free whole-genome sequence data. Genome Res. 2017 Nov;27(11):1895-1903. doi: 10.1101/gr.225672.117. Epub 2017 Sep 8. PMID: 28887402; PMCID: PMC5668946.

Grassano M, Calvo A, Moglia C, Brunetti M, Barberis M, Sbaiz L, Canosa A, Manera U, Vasta R, Corrado L, D'Alfonso S, Mazzini L, Scholz SW, Dalgard C, Ding J, Gibbs RJ, Chia R, Traynor BJ, Chiò A; American Genomic Center. Mutational Analysis of Known ALS Genes in an Italian Population-Based Cohort. Neurology. 2021 Jan 26;96(4):e600-e609. doi: 10.1212/WNL.0000000000011209. Epub 2020 Nov 18. PMID: 33208543; PMCID: PMC7905787.

Grassano M, Calvo A, Moglia C, Sbaiz L, Brunetti M, Barberis M, Casale F, Manera U, Vasta R, Canosa A, D'Alfonso S, Corrado L, Mazzini L, Dalgard C, Karra R, Chia R, Traynor B, Chiò A. Systematic evaluation of genetic mutations in ALS: a population-based study. J Neurol Neurosurg Psychiatry. 2022 Jul 27;93(11):1190–3. doi: 10.1136/jnnp-2022-328931. Epub ahead of print. PMID: 35896380; PMCID: PMC9606529.

Montuschi A, Iazzolino B, Calvo A, Moglia C, Lopiano L, Restagno G, Brunetti M, Ossola I, Lo Presti A, Cammarosano S, Canosa A, Chiò A. Cognitive correlates in amyotrophic lateral sclerosis: a population-based study in Italy. J Neurol Neurosurg Psychiatry. 2015 Feb;86(2):168-73. doi: 10.1136/jnnp-2013-307223. PMID: 24769471.

Poletti B, Aiello EN, Solca F, Torre S, Carelli L, Ferrucci R, Verde F, Ticozzi N, Silani V. Diagnostic properties of the Italian ECAS Carer Interview (ECAS-CI). Neurol Sci. 2023 Mar;44(3):941-946. doi: 10.1007/s10072-022-06505-x. PMID: 36417015.

Poletti B, Solca F, Carelli L, Madotto F, Lafronza A, Faini A, Monti A, Zago S, Calini D, Tiloca C, Doretti A, Verde F, Ratti A, Ticozzi N, Abrahams S, Silani V. The validation of the Italian Edinburgh Cognitive and Behavioural ALS Screen (ECAS). Amyotroph Lateral Scler Frontotemporal Degener. 2016 Oct-Nov;17(7-8):489-498. doi: 10.1080/21678421.2016.1183679. Epub 2016 May 24. PMID: 27219526.

Renton AE, Majounie E, Waite A, Simón-Sánchez J, Rollinson S, Gibbs JR, Schymick JC, Laaksovirta H, van Swieten JC, Myllykangas L, Kalimo H, Paetau A, Abramzon Y, Remes AM, Kaganovich A, Scholz SW, Duckworth J, Ding J, Harmer DW, Hernandez DG, Johnson JO, Mok K, Ryten M, Trabzuni D, Guerreiro RJ, Orrell RW, Neal J, Murray A, Pearson J, Jansen IE, Sondervan D, Seelaar H, Blake D, Young K, Halliwell N, Callister JB, Toulson G, Richardson A, Gerhard A, Snowden J, Mann D, Neary D, Nalls MA, Peuralinna T, Jansson L, Isoviita VM, Kaivorinne AL, Hölttä-Vuori M, Ikonen E, Sulkava R, Benatar M, Wuu J, Chiò A, Restagno G, Borghero G, Sabatelli M; ITALSGEN Consortium; Heckerman D, Rogaeva E, Zinman L, Rothstein JD, Sendtner M, Drepper C, Eichler EE, Alkan C, Abdullaev Z, Pack SD, Dutra A, Pak E, Hardy J, Singleton A, Williams NM, Heutink P, Pickering-Brown S, Morris HR, Tienari PJ, Traynor BJ. A hexanucleotide repeat expansion in C9ORF72 is the cause of chromosome 9p21-linked ALS-FTD. Neuron. 2011 Oct 20;72(2):257-68. doi: 10.1016/j.neuron.2011.09.010. Epub 2011 Sep 21. PMID: 21944779; PMCID: PMC3200438.

**eTable 1**. Cognitive tests: explored domains

| **Domains** | **Tests** |
| --- | --- |
| Executive functions | Letter Fluency test (FAS) |
|  | Category Fluency Test (CAT) |
|  | Trail Making Test B-A (TMT B-A) |
|  | Frontal Assessment Battery (FAB) |
|  | ECAS Executive Function score |
|  | ECAS Verbal Fluency score |
| Verbal memory | Rey Auditory Verbal Learning Test, Immediate Recall (RAVL-IR) |
|  | Rey Auditory Verbal Learning Test, delayed Recall (RAVL-DR) |
|  | Babcock Story Recall Test, Immediate Recall (BSRT-IR) |
|  | Babcock Story Recall Test, Delayed Recall (BSRT-DR) |
|  | ECAS Memory score |
| Language | Token test (up to 2016) |
|  | Battery for the Analysis of Aphasic Deficits (semantic systems tests 7 and 8) (up to 2016) |
|  | Boston Naming Test (from 2016) |
|  | ECAS Language score (from 2016) |
| Visual Memory | Rey-Osterrieth Complex Figure Test, differed recall (ROCF-DR) |
| Visuoconstructive abilities | Rey-Osterrieth Complex Figure Test, Immediate Recall (ROCF-IR) |
|  | Clock Drawing Test (Clock) |
|  | ECAS Visuospatial Abilities score |
| Attention/working memory | Digit Span Forward (FW) |
|  | Digit Span Backward (BW) |
| Psychomotor speed | Trail Making Test A (TMT A) |
| Fluid intelligence | Raven’s Colored Progressive Matrices (CPM47) |
| Cognitive flexibility | Trail Making Test B (TMT B) |
| Theory of mind (social cognition) | Story-based Empathy Task (SET) (from 2018) |
| Behavior | Frontal Systems Behavior Scale (FrSBe) |
|  | ECAS Behavior score |
|  | Frontal Behavioural Inventory (FBI) |
| Non-ALS | Mini Mental State Examination (MMSE) |

**eTable 2**. References of normative data of used tests

| **Test** | **Italian normative reference** |
| --- | --- |
| MMSE | Carpinelli Mazzi M, Iavarone A, Russo G, et al. Mini-Mental State Examination: new normative values on subjects in Southern Italy. Aging Clin Exp Res. 2020;32(4):699-702. doi:10.1007/s40520-019-01250-2 |
| FAS | Caltagirone C, Gainotti G, Carlesimo GA, et al. Batteria per la valutazione del Deterioramento Mentale (parte I): descrizione di uno strumento di diagnosi neuropsicologica. Archivio Di Psicologia, Neurologia E Psichiatria 1995; 56(4), 461-470 |
| CAT | Spinnler H, Tognoni G. Standardizzazione e taratura italiana di test neuropsicologici. Ital J Neurol Sci. 1987; 6 [Suppl. 8]: 78-80 |
| FAB | Appollonio I, Leone M, Isella V, et al. The frontal assessment battery (FAB): Normative values in an Italian population sample. Neurol Sci. 2005;26(2):108-116. doi:10.1007/s10072-005-0443-4 |
| Digit Span FW and BW | Monaco M, Costa A, Caltagirone C, Carlesimo GA. Forward and backward span for verbal and visuo-spatial data: Standardization and normative data from an Italian adult population. Neurol Sci. 2013;34(5):749-754. doi:10.1007/s10072-012-1130-x |
| TMT A, B and B-A | Siciliano M, Chiorri C, Battini V, Sant'Elia V, Altieri M,v Trojano L, Santangelo G. Regression-based normative data and equivalent scores for Trail Making Test (TMT): an updated Italian normative study. Neurol Sci. 2019 Mar;40(3):469-477. doi: 10.1007/s10072-018-3673-y. |
| RAVL-IR and IR | Caltagirone C, Gainotti G, Carlesimo GA, et al. Batteria per la valutazione del Deterioramento Mentale (parte I): descrizione di uno strumento di diagnosi neuropsicologica. Archivio di Psicologia, Neurologia e Psichiatria 1995; 56(4), 461-470 |
| BSRT-IR and DR | Carlesimo GA, Buccione I, Fadda L, et al. Normative data of two memory tasks: Short-Story recall and Rey’s Figure. Nuova Riv di Neurol 2002;12(1):1-13. |
| ROCF-IR and DR | Carlesimo GA, Buccione I, Fadda L, et al. Normative data of two memory tasks: Short-Story recall and Rey’s Figure. Nuova Riv di Neurol 2002;12(1):1-13. |
| CPM47 | Caltagirone C, Gainotti G, Carlesimo GA, et al. Batteria per la valutazione del Deterioramento Mentale (parte I): descrizione di uno strumento di diagnosi neuropsicologica. Archivio di Psicologia, Neurologia e Psichiatria 1995; 56(4), 461-470 |
| ECAS | Poletti B, Solca F, Carelli L, Madotto F, Lafronza A, Faini A, Monti A, Zago S, Calini D, Tiloca C, Doretti A, Verde F, Ratti A, Ticozzi N, Abrahams S, Silani V. The validation of the Italian Edinburgh Cognitive and Behavioural ALS Screen (ECAS). Amyotroph Lateral Scler Frontotemporal Degener. 2016 Oct-Nov;17(7-8):489-498. doi: 10.1080/21678421.2016.1183679. |
| SET | Dodich A, Cerami C, Canessa N, Crespi C, Iannaccone S, Marcone A, Realmuto S, Lettieri G, Perani D, Cappa SF. A novel task assessing intention and emotion attribution: Italian standardization and normative data of the Story-based Empathy Task. Neurol Sci. 2015 Oct;36(10):1907-12. doi: 10.1007/s10072-015-2281-3. Epub 2015 Jun 14. PMID: 26072203. |

**eTable 3**. List of ALS cases with ANXA11 variants

| DNA code | Genomic variant | Protein | Gene domain | Sex | Age at onset (years) | Site of onset | FALS | Cognition | KING’s at diagnosis | MiToS at diagnosis |
| --- | --- | --- | --- | --- | --- | --- | --- | --- | --- | --- |
| COD-0067 | c.7A>G | p.Y3H | LCD | F | 75 | B | N | ALSci | 2 | 0 |
| PAR-2011-106 | c.102G>A | p.M34I | LCD | M | 60 | LL | N | ALSci | 3 | 1 |
| PAR-2015-020 | c.102G>A | p.M34I | LCD | M | 69 | LL | N | ALSci | 1 | 0 |
| PAR-2015-114 | c.103C>T | p.P35S | LCD | M | 70 | LL | N | ALS-FTD | 3 | 1 |
| PAR-2010-061 | c.119A>G | p.D40G | LCD | F | 55 | B | Y | ALS-FTD | 1 | 0 |
| PAR-2015-033 | c.119A>G | p.D40G | LCD | F | 62 | LL | N | ALS-FTD | 1 | 1 |
| COD-2256 | c.137C>T | p.A46V | LCD | M | 75 | LL | N | n/a | 2 | 0 |
| PAR-2013-089 | c.173C>T | p.A58V | LCD | M | 78 | LL | N | ALSbi | 2 | 1 |
| PAR-2010-130 | c.477_503del | p.159_168del | LCD | M | 64 | LL | N | ALScbi | 2 | 0 |
| PAR-2009-070 | c.731C>T | p.T244M | N-LCD | M | 63 | UL | N | ALS-FTD | 1 | 0 |
| COD-2530 | c.739G>A | p.G247S | N-LCD | M | 70 | LL | N | ALSci | 3 | 0 |
| PAR-2011-104 | c.905G>A | p.R302H | N-LCD | F | 76 | B | Y | n/a | 1 | 0 |
| PAR-2015-016 ^ | c.823G>A | p.R308X | N-LCD | M | 78 | B | Y | ALS-FTD | 3 | 0 |
| PAR-2012-082 ° | c.1010A>T | p.L337H | N-LCD | F | 78 | B | N | n/a | 2 | 0 |
| PAR-2014-048 | c.1191G>C | p.E397D | N-LCD | F | 45 | LL | N | ALSbi | 2 | 1 |
| COD-1749 | c.1208G>C | p.G403A | N-LCD | M | 61 | LL | N | n/a | 3 | 1 |
| NOV20-15 * | c.1243T>A | p.S415T | N-LCD | M | 63 | B | Y | ALSci | 1 | 0 |
| COD-1741 | c.1424G>A | p.R475Q | N-LCD | M | 82 | B | N | ALS-FTD | 3 | 1 |

* This patient carried also a *C9ORF72* pathogenic expansion

^ This patient carried also the p.E581G variant in the *ERBB4* gene

° This patient carried also the p.S687C variant of the *EPHA4* gene

LCD, low complexity domain; N-LCD, non-low complexity domain. ALSci, patients with isolated cognitive impairment: ALSbi, patients with isolated behavioral impairment; ALScbi, patients with both cognitive and behavioral impairment; ALS-FTD. patients with frontotemporal dementia; n/a, not available. B, bulbar onset; LL, lower limb onset; UL, upper limb onset

**eTable 4**. Pathogenicity prediction for *ANXA11* (NM_001157) variants

| **Protein position** | **Amino acid** | **ClinVar** | **VarSome** | **SIFT** | **PolyPhen** | **CADD** | **gnomAD NFE MAF** | **Mutation Taster** | **PROVEAN** | **fathmm-MKL** |
| --- | --- | --- | --- | --- | --- | --- | --- | --- | --- | --- |
| 3 | Y/H | - | LB | Del | - | 24.1 | 2.86E-06 | Dis | Neu | Tol |
| 34 | M/I | - | VUS | Tol | - | 17.15 | 9.96E-05 | DIs | Neu | Dam |
| 35 | P/S | VUS | VUS | Tol | - | 18.13 | 7.38E-06 | Dis | Neu | Dam |
| 40 | D/G | LP | LP | Tol | - | 21.5 | 1.86E-06 | Dis | Neu | Dam |
| 46 | A/V | VUS | VUS | Del | - | 23.3 | 1.03E-05 | Pol | Neu | Dam |
| 58 | A/V | VUS | VUS | Tol | - | 25.8 | 8.99E-06 | Dis | Neu | Dam |
| 159-168 | PLPGQQQPVP/P | - | - | - | - | - | - | - | - | - |
| 244 | T/M | LB | LB | Del | Pro D | 32 | 4.23E-03 | Dis | Dam | Dam |
| 247 | G/S | VUS | VUS | Del | Pro D | 29.4 | 2.71E-06 | Dis | Dam | Dam |
| 302 | R/H | B | B | Del | Pro D | 34 | 7.91E-05 | Dis | Dam | Dam |
| 308 | R/* | VUS | VUS | Del | - | 42.0 | 1.44E-05 | - | - | Tol |
| 337 | L/H | - | B | Del | Pos D | 31.0 | 5.05E-04 | Dis | Dam | Dam |
| 397 | E/D | VUS | VUS | Del | Pro D | 21.1 | 5.71E-06 | Dis | Dam | Dam |
| 403 | G/A | - | LB | Del | Pos D | 24.6 | 2.86E-06 | Pol | Dam | Dam |
| 415 | S/T | VUS | VUS | Del | Pos D | 26.4 | 3.60E-06 | Dis | Dam | Dam |
| 475 | R/Q | VUS | VUS | Tol | Pos D | 25.1 | 5.58E-05 | Dis | Neu | Dam |

NFE: Non-Finnish Europeans; MAF: Minor Allele Frequency

Dam: Damaging; Del: Deleterious; Dis: Disease Causing; LB, Likely Benign; LP; Likely pathogenic; Neu: Neutral; Pol: Polymorphism; Pos D: Possibily Damaging; Pro D: Probably Damaging; Tol: Tolerated; VUS: Variant of Uncertain Significance

**eFigure 1**. Flow-chart of the study


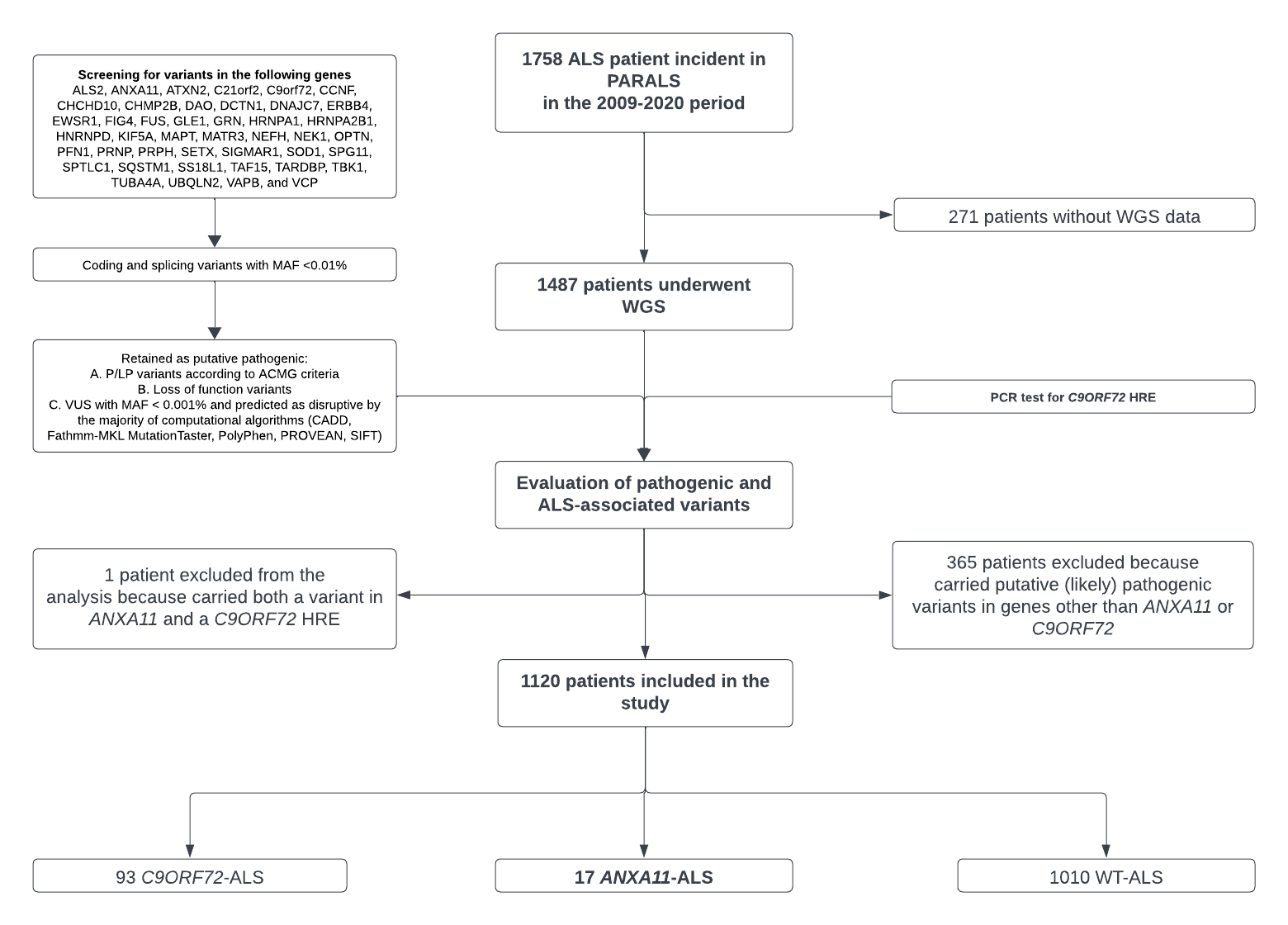


366 patients excluded because carried putative (likely) pathogenetic variants in gene other than *ANXA11* or *C9ORF72*

1121 patients included in the study

18 *ANXA11* variants, 4 of which were classified as a benign/likely benign
